# AOGS in 2025: Opportunities and challenges

**DOI:** 10.1111/aogs.15044

**Published:** 2024-12-22

**Authors:** Amarnath Bhide

**Affiliations:** ^1^ Fetal Medicine Unit St. George's Hospital and City St. George's, University of London London UK; ^2^ Baylor College of Medicine Waco Texas USA

After 10 years at the helm, Professor Ganesh Acharya has passed on the mantle of chief editorship of AOGS. Under his leadership the journal has gone from strength to strength. I am excited and intimidated in equal measures to step up as the 13th Chief Editor and the first one from outside Scandinavia. Although the team is not new to me—I have been a Deputy Chief Editor of AOGS for the last 6 years—the responsibility and expectations are certainly new. I am aware that I would be standing on the shoulders of giants.

In his editorial last month (AOGS‐24‐1372.R1),^1^ Professor Acharya looked back at his time at AOGS. The journal successfully transitioned to open access publishing, maintained its ranking in the O & G journals, maintained the reputation for publishing reliable, good quality research in our specialty, and strengthened the reputation for quick and effective decision making for the submitted manuscripts. He outlined our principles—excellence, fairness, openness, and equality, that he has eschewed and I hope to emulate.

We are experiencing a marked increase in the number of submissions. Last year (2023) we dealt with 1185 submitted manuscripts, this year we are already past 1500. This makes editorial work challenging. With the support of the Nordic Federation of Societies of Obstetrics and Gynecology (NFOG), we now have two Deputy Chief Editors instead of one. I am looking forward to working with them.

We have been on the receiving end of the wrath of some of our authors for transitioning to open‐access publishing. In their view, we were becoming no different from “predatory journals” that have mushroomed over the last few years. A list of such journals (beallslist.net) is available to view on the internet, and I am proud to note that AOGS does not figure on it. The Directory of Open Access Journals (DOAJ, www.doaj.org) was developed in response to discussions about predatory publishing. The decision to transform to open access publishing was that of NFOG rather than our publishers. The decision did not stem from profit making. On the contrary, the NFOG was of the opinion that research that benefits mankind should not be hidden behind paywalls and should be accessible to all. Governments of Scandic nations—bar the exception of Denmark and Iceland—support open access publishing by entering into transformational agreements with the publishers, so that the publishing charges are not borne by either the authors or the readers. However, several of our competitors are still publishing in a hybrid format and not fully open access. Another publishing model is “diamond access,” where the authors do not pay publication charges but the manuscripts are still free to view. Many such journals are published by university departments and publication charges are borne by their internal budgets or through charity support. This model has been successfully used in South America.^2^ Unfortunately, some journals have struggled at times with the sustainability of financial support. We have not found a solution to the vexatious problem of how best to fund academic publishing.

Recently, we have witnessed concerns regarding the possibility of scientific misconduct in published research,^3^ some of which were published in AOGS. Although researchers have high integrity in the vast majority and we take every precaution to avoid this from happening, rare unscrupulous elements do exist. Availability of tools taking advantage of advances in artificial intelligence has the potential to make the problem of finding the culprits and proving their misdemeanor increasingly difficult. Therefore, we endorse collaborative efforts with leading scientific journals in our field to uphold scientific integrity in publishing.^4^ It takes years to build up a good reputation but a very short time to lose it. A checklist for assessing the trustworthiness of a randomized controlled trial has already been published.^5^ One may not agree with every element proposed by the authors, but it is better to start somewhere than not start at all.

Advances in information technology, computing and artificial intelligence (AI) are looked upon with doubt and intimidation by many. Although capable of causing a big dis‐service in the wrong hands, I welcome the technology with open arms, eyes and mind. Whether we like it or not, AI is here to stay. It is up to us either to shun it or to embrace it. I am all for improvements in user experience, and if AI helps with this, why should one not use it? With this in mind, we plan to harness some of the power of AI to generate podcasts for selected papers published in AOGS and make it available to our readers/listeners. I am told that the way the younger generation learns is changing. Trial of learning by listening to a podcast as opposed to reading a scientific article would be interesting. Our experiment will tell us if this trial is a success or waste of our social media editor's time.

There are challenges ahead as well as exciting opportunities. The future is likely to depend on how we respond to changes in the field of information acquisition, processing, and interpretation. Our mission is not only to spread information or impart knowledge to our readers but to make them wiser. In the words of the 18th century English preacher Charles Spurgeon,^5^ “Wisdom is the right use of knowledge. To know is not to be wise. Many men know a great deal, and are all the greater fools for it. There is no fool so great a fool as a knowing fool. But to know how to use knowledge is to have wisdom.”

## AUTHOR CONTRIBUTIONS

Conception and writing: Amarnath Bhide.

## ETHICS STATEMENT

Not applicable.
